# Predicting high-cost care in a mental health setting

**DOI:** 10.1192/bjo.2019.96

**Published:** 2020-01-17

**Authors:** Craig Colling, Mizanur Khondoker, Rashmi Patel, Marcella Fok, Robert Harland, Matthew Broadbent, Paul McCrone, Robert Stewart

**Affiliations:** Applied Clinical Informatics Lead, SLaM Biomedical Research Center, South London & Maudsley Foundation NHS Trust, UK; Senior Lecturer in Medical Statistics, University of East Anglia, Norwich Medical School, UK; MRC UKRI Health Data Research UK Fellow, Department of Psychosis Studies, Institute of Psychiatry, Psychology and Neuroscience, Kings College London; and South London & Maudsley Foundation NHS Trust, UK; Visiting Researcher, Psychological Medicine, Institute of Psychiatry, Psychology and Neuroscience, Kings College London; and Central and North West London NHS Foundation Trust, UK; Clinical Director of Psychosis, Psychosis CAG, South London & Maudsley Foundation NHS Trust, UK; Informatics Lead, SLaM Biomedical Research Center, South London & Maudsley Foundation NHS Trust, UK; Professor of Health Economics, School of Health Science, Institute of Psychiatry, Psychology and Neuroscience, Kings College London, UK; Professor of Psychiatric Epidemiology and Clinical Informatics, Psychological Medicine, Institute of Psychiatry, Psychology and Neuroscience, Kings College London; and South London & Maudsley Foundation NHS Trust, UK

**Keywords:** Digital health records, mental health service, prediction, natural language processing

## Abstract

**Background:**

The density of information in digital health records offers new potential opportunities for automated prediction of cost-relevant outcomes.

**Aims:**

We investigated the extent to which routinely recorded data held in the electronic health record (EHR) predict priority service outcomes and whether natural language processing tools enhance the predictions. We evaluated three high priority outcomes: in-patient duration, readmission following in-patient care and high service cost after first presentation.

**Method:**

We used data obtained from a clinical database derived from the EHR of a large mental healthcare provider within the UK. We combined structured data with text-derived data relating to diagnosis statements, medication and psychiatric symptomatology. Predictors of the three different clinical outcomes were modelled using logistic regression with performance evaluated against a validation set to derive areas under receiver operating characteristic curves.

**Results:**

In validation samples, the full models (using all available data) achieved areas under receiver operating characteristic curves between 0.59 and 0.85 (in-patient duration 0.63, readmission 0.59, high service use 0.85). Adding natural language processing-derived data to the models increased the variance explained across all clinical scenarios (observed increase in *r*^2^ = 12–46%).

**Conclusions:**

EHR data offer the potential to improve routine clinical predictions by utilising previously inaccessible data. Of our scenarios, prediction of high service use after initial presentation achieved the highest performance.

Mental healthcare is costly, recent estimates suggesting that direct costs in England are around £22.5 billion per year.^1,2^ In-patient care is a particularly costly aspect of mental healthcare: during 2010/11, investment in in-patient care within England for adults aged between 18 and 64 was estimated at £2 billion.^1^ Although knowledge of average costs is important, it is recognised that these vary substantially between patients, with a small number accounting for disproportionate resources.^3^ The mean duration of finished consultant in-patient episodes in England during 2016/17 was 51 days and the median duration was 19 days.^4^ Recent estimates suggest common mental disorders have a prevalence rate of around one adult in six (15.7%).^5^ Prediction of high-cost care is clearly important for identifying avoidable reasons. However, previous predictive studies in the UK have used research data from small samples^6,7^ or predictions were restricted to data limited in scope.^8,9^ The increasing availability of routine data from detailed electronic records potentially allows a more robust investigation of variations in cost and predictors of this.

This study aimed to investigate the extent to which high priority service outcomes might be predicted by data routinely recorded within the electronic health record (EHR). We sought to take advantage of potentially high volumes of in-depth text-mined information for predictive modelling that has remained hitherto untapped. We investigated, three different service outcomes that might benefit from predictive models: (a) extended duration of mental health hospital admissions, (b) mental health hospital readmission following in-patient discharge, and (c) high-intensity service use following first referral to mental healthcare. We used a clinical database derived from the EHR of a large mental healthcare provider that includes metadata derived through natural language processing information extraction techniques.^10,11^

## Method

### Setting

The study was carried out in the South London & Maudsley NHS Foundation Trust (SLaM): a secondary mental healthcare provider that serves a population of 1.2 million residents in four London boroughs (Lambeth, Southwark, Lewisham and Croydon). Electronic records have been used comprehensively across all SLaM services since 2006 and the Clinical Record Interactive Search (CRIS) system, developed in 2008, allows searching and retrieval of anonymised real-time information from SLaM's EHR, with over 400 000 patients currently represented. The development and protocol of this case register have been described in detail elsewhere.^10,11^

The predictor variables were assembled based on candidates listed in previous reviews^6–9,12^ and following project meetings that had representation from psychiatrists, researchers, informaticians and National Health Service (NHS) managers. Structured data from the EHR included patient demographics, service use, and health and social functioning measured by the Health of the Nation Outcome Scales. In addition, natural language processing techniques for information extraction^13^ have been developed in CRIS to derive a range of structured data from unstructured free-text fields, including mentions of medication use and symptoms.^11^ Symptom extraction comprises over 50 individual natural language processing algorithms that can be broadly categorised into the following high-level domains: catatonic, depressive, disorganisation, manic, negative and positive.^14^ These were developed using TextHunter,^15^ which is a tool that facilitates the rapid development and deployment of general architecture for text engineering^16^ machine learning applications with an inbuilt annotation tool to enable the creation of development and gold standard samples.

As described, three outcomes were evaluated, chosen and defined pragmatically on the basis of local trust priorities and potential tractability for predictive model development and evaluation. The first two focused on severe mental illness diagnostic categories on the assumption that in-patient care outcomes might have heterogeneous determinants between different diagnostic groups, whereas the aim of the third study did not restrict by diagnosis because diagnoses are often unclear shortly after first presentation and it was envisaged that a prediction algorithm would have more utility when diagnosis-agnostic. Each model was constructed around an *a priori* ‘index date’ at which point multiple measures would be defined up to that date and used to predict a subsequent chosen outcome. The rationale for this was that an algorithm might thereby be generated that could at least potentially be run automatically from an EHR in order to feedback relevant outcome probabilities in routine clinical care. Predictive model evaluations followed similar steps and algorithms for all three outcomes: developing and optimising models for events in one calendar year and then evaluating them in the subsequent year. The specific modelling details for each outcome are described in the following sections.

### Outcome 1 – predicting extended duration of hospital admissions

#### Sample definition

Using CRIS, we selected patients aged between 18 and 65 on admission with a psychotic disorder (defined as receiving an International Classification of Diseases (ICD)-10 F20-29 or F31 diagnosis within the previous 12-month period), who had been admitted for at least 7 days to a general acute ward, psychiatric intensive care unit (PICU) or triage in-patient service. If a patient had more than one recorded admission initiated during the time frame, the first one was selected as the index admission.

#### Index date, predictors and outcome

We defined an index date of 7 days into in-patient care (i.e. restricting the sample to those with at least 7-day admissions) and sought to predict in-patient care lasting 76 days or more following that date; this was the upper quartile of the distribution in the development sample. Predictor data were obtained from EHRs within the 12-month period prior to the index date; 88 predictor variables were used, listed in supplementary Table 1 (available at https://doi.org/10.1192/bjo.2019.96).

#### Prediction model development and evaluation

We used in-patient admissions over two separate calendar years to create a development sample (admissions during 2012) within which to train the predictive algorithm and a validation sample (admissions during 2013) for final evaluation of its performance. The development sample comprised 808 eligible patients.

### Outcome 2 – predicting readmission following discharge from in-patient care

#### Sample definition

Using CRIS, we selected patients aged between 18 and 65 with a psychotic disorder (defined as receiving an ICD-10 F20-29 or F31 diagnosis within the previous 12-month period), who had been discharged from a general acute ward, PICU or triage in-patient service. If a patient had more than one discharge during the time frame, the first one was selected.

#### Index date, predictors and outcome

We defined the index date as the in-patient discharge date and sought to predict in-patient readmission within the subsequent 90 days. Predictor data were obtained up to 1 year prior to the index date; 107 predictor variables were used, listed in supplementary Table 2.

#### Prediction model development and evaluation

We used in-patient discharges over two separate calendar years to create a development sample (discharges during 2012) within which to train the predictive algorithm, and a validation sample (discharges during 2013) for final evaluation of its performance. The development sample comprised 1650 eligible patients.

### Outcome 3 – predicting high-intensity service use following first referral

#### Sample definition

We selected patients following their first presentation to SLaM services who were aged 18 years and over at acceptance on a given team's case-load. Presentations to addictions services and patients who had less than 90 days' service duration were excluded.

#### Index date, predictors and outcome

The index date was defined as 90 days following the patient's first presentation to a SLaM service, and we sought to predict highest-decile service cost over the 12 months following that date; this amounted to £2832 or more per year. The total service cost was based on the reference cost of a mental health bed day and a contact with a mental specialist team specified within the annual compendium of unit costs produced by the University of Kent^17^ multiplied by the number of in-patient bed days and out-patient service contacts within the 12-month period following the index date. Predictor data were obtained for the 90 days following the date of the first presentation to service; 93 predictor variables were used, listed in supplementary Table 3.

#### Prediction model development and evaluation

We used first presentations over two separate calendar years to create a development sample (first presentations during 2012) within which to train the predictive algorithm, and a validation sample (first presentations during 2013) for final evaluation of its performance. The development sample comprised 4494 eligible patients.

### Statistical analysis

All analyses used R version 3.2.1. We used a multivariable logistic regression model to build the prediction models. During model development, variables that did not appear to make a sufficient contribution in predicting the outcome (defined as those with *P*-values >0.5 for respective regression coefficients in univariate analyses) were dropped. Outliers with absolute standardised residuals >3.0 were also removed. We used generalised variance inflation factor (GVIF) for identifying multicollinearity.^18^ Independent variables with an GVIF^1/2d.f.^ (i.e. GVIF to the power 1/2d.f.) value more than 3.0, where d.f. is the number of coefficients for the variable in question, were excluded. The remaining variables were entered into a multivariable logistic regression analysis using Akaike's information criterion (AIC)-based stepwise variable selection to derive a model with the greatest predictive utility. The predictive power of the model was tested using fivefold cross-validation in order to determine the accuracy of prediction.

The performances of the prediction models on the development and validation samples were evaluated using receiver operating characteristic (ROC) curves, calculation of the area under the ROC curves (AUCs) and testing the difference between two AUCs using DeLong's test.^19^ The variables that contributed to the prediction were ranked based on the extent of their contribution measured by the absolute *z*-value.

Secondary analyses were undertaken to test the extent to which natural language processing-derived variables contributed to the overall predictability of the models by calculating the McFadden pseudo *r*^2^ for a logistic regression model.^20^ To test the relative quality of the model the AIC for the full model was compared with the model without the natural language processing-derived data and the percentage reduction in AIC calculated.

## Results

### Outcome 1 – predicting extended duration of hospital admissions

The development and validation samples were similar in size and with respect to demographic and service characteristics (supplementary Table 4). Within the validation sample, those with extended duration of hospital admissions were more likely to be detained under Section 3 of the Mental Health Act (*P* < 0.001).

The prediction models for the extended hospital duration within the development sample achieved a moderate AUC (0.77) with sensitivity of 0.63 and specificity of 0.80 at an optimal cut-off; within the validation sample, the AUC was 0.63, and optimum sensitivity and specificity were 0.63 and 0.59, respectively. Supplementary Table 5 describes all variables within the final model and their individual contribution to the prediction.

Detention under Section 3 of the Mental Health Act at the index date was the most predictive variable (*z*-score 5.68, odds ratio (OR) = 4.05, 95% CI 2.51–6.60). The natural language processing-derived data better explained the variance in the model by 40% from 0.11 to 0.18 when tested using the McFadden pseudo *r*^2^ statistic, and reduced the AIC from 832.74 to 798.01: a relative reduction of 4.2% (χ^2^ = 70.729 (d.f. = 18), *P* < 0.001).

In a further exploratory analysis, we stratified the sample by the most predictive variable, Mental Health Act status at index date, into three groups: (a) informal status, (b) Section 2, (c) Section 3. The development data-sets had 320, 294 and 174 patients, respectively, and it was concluded that the sample for Section 3 was too small to proceed with. The prediction model for extended hospital duration in the sample with Section 2 status performed moderately within the development sample (AUC 0.81, sensitivity 0.85, specificity 0.67) but poorly within the validation sample (AUC 0.52, sensitivity 0.77, specificity 0.33). The prediction models for the extended hospital duration in the sample with informal status were also moderate within the development sample (AUC 0.76, sensitivity 0.80, specificity 0.60) and poor within the validation sample (AUC 0.51, sensitivity 0.41, specificity 0.68).

### Outcome 2 – predicting readmission following discharge from mental health in-patient care

The development and validation samples were similar in size. The development sample had fewer patients from ‘other’ ethnic background (*P* = 0.043) and with a bipolar disorder (*P* = 0.021) diagnosis. Within the validation sample, there were no significant differences in demographic or service characteristics for those readmitted. The descriptions of the development and validation samples are available in supplementary Table 6.

The prediction models for hospital readmission within the development sample achieved a moderate to strong AUC (0.84) with sensitivity of 0.67 and specificity of 0.85 at an optimal cut-off, but this performance was substantially reduced within the validation sample (AUC 0.59, sensitivity 0.47, specificity 0.75). Supplementary Table 7 describes all variables within the final model and their individual contribution to the prediction. The number of emergency admissions in the previous 12 months prior to in-patient discharge was the most predictive variable (*z* score 3.52, OR = 1.93, 95% CI 1.33–2.78). The natural language processing-derived data increased the predictability of the model by 46.4% from 0.17 to 0.31 when tested using the McFadden pseudo *r*^2^ statistic, and reduced the AIC from 689.3 to 655.6, a relative reduction of 4.9% (χ^2^ = 71.63 (d.f. = 19), *P* < 0.001).

### Outcome 3 – high-intensity service use following first referral

The development and validation samples were broadly consistent in terms of the overall sample size. The development sample had more patients from a White ethnic background (*P* = 0.019), with an organic diagnosis (*P* < 0.001), or with a personality disorder (*P* = 0.030) or substance misuse (*P* = 0.007) diagnosis. Within the validation sample, patients with high-costs were younger (*P* = 0.011), were more likely to be divorced or single (*P* < 0.001), from a Black ethnic background (*P* < 0.001) and to have their most recent diagnosis categorised as a mood/anxiety disorder (*P* < 0.001) or schizophrenia (*P* < 0.001). The descriptions of the development and validation samples are available in supplementary Table 8.

The prediction models for the total service cost achieved a moderate to strong AUC (0.87) within the development sample, with sensitivity of 0.81 and specificity of 0.78 at an optimal cut-off. Within the validation sample, this performance was largely maintained (AUC 0.85, sensitivity 0.67, specificity 0.86). Supplementary Table 9 describes all variables within the final model and their individual contribution to the prediction. Service use variables such as in-patient bed days (*z* score 8.45, OR = 1.04, 95% CI 1.03–1.05) and community contact (*z*-score 6.35, OR = 1.11, 95% CI 1.08–1.15) were the most predictive. The natural language processing-derived data increased the predictability of the model by 12.1% from 0.29 to 0.33 when tested using the McFadden pseudo *r*^2^ statistic, and reduced the AIC from 2106.7 to 2032.1, a relative reduction of 3.5% (χ^2^ = 126.25 (d.f. = 26), *P* < 0.001).

### Outcomes 1–3 compared

**Fig. 1:**
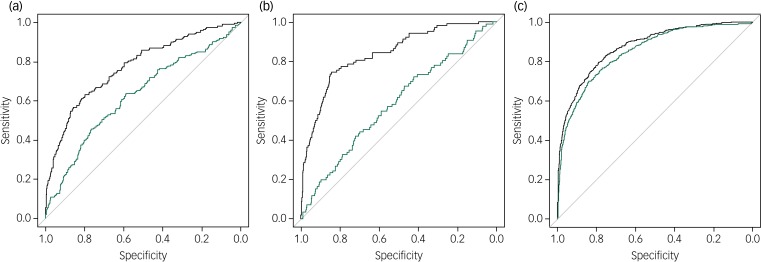
(a) Receiver operating characteristic (ROC) curve of model performance for extended duration of hospital admission. (b) ROC curve of model performance for hospital readmission. (c) ROC curve of model performance for high total service cost.

Table 1 describes the results for all clinical outcomes evaluated and ROC analyses are displayed in Fig. 1. As described, development models achieved best performance levels (*R*^2^ and AUC) for outcomes 2 and 3, but only outcome 3 sustained satisfactory performance in its validation setting.

**Table 1:** Model performance for all clinical scenarios explored

Clinical scenario and primary outcome	Development sample				Validation sample		
	Sensitivity	Specificity	*R*^2^	AUC	Sensitivity	Specificity	AUC
1. Length of stay, predicting highest 25% hospital admission duration	0.63	0.80	0.18	0.77	0.63	0.59	0.63
1(a) Length of stay: MHA Section 2, 1st decile hospital admission duration	0.85	0.67	0.22	0.81	0.77	0.33	0.52
1(b) Length of stay: informal, 1st decile hospital admission duration	0.80	0.60	0.17	0.76	0.41	0.68	0.51
2. Predicting hospital readmission within 90 days	0.67	0.85	0.30	0.84	0.47	0.75	0.59
3. Total service cost, predicting highest 10% service cost within 12 months	0.81	0.78	0.31	0.87	0.86	0.67	0.85

AUC, area under receiver operating characteristic curve; MHA Mental Health Act.

The black line represents the performance for the development sample and the green line represents the performance for the validation sample.

## Discussion

### Main findings

We used a large clinical data resource to investigate the extent to which three different outcomes might be predicted in routine practice from routinely recorded data in the mental healthcare EHR. In all three outcomes, models were developed and optimised within one calendar year and their predictive ability evaluated in the next year. In the first two case studies evaluated, for extended (highest quartile) duration of hospital admission and readmission (within 90 days) following hospital admission, predictive models did not achieve adequate levels of performance when evaluated in independent data-sets. However, the third case study, predicting highest 12-month service costs 90 days into a first referral episode, showed promisingly sustained performance from development to evaluation data.

### Use of natural language processing

The digitisation of health records has created unprecedented volumes of information derived from routine care with high potential to transform the way in which services are delivered and tailored to the individual. In particular, programming capability and computer capacity are likely now to be at a sufficient stage of development to support the generation of algorithms to inform clinician and/or service decision-making based on real-time ‘big data’ derived from the EHR. However, this depends on both the availability, accuracy and quality of information recorded, as well as on achieving pipelines and platforms for information processing and delivery. In mental healthcare, a key challenge has been the fact that most clinically valuable information is recorded in text fields within the EHR, such as case notes and correspondence, rather than as structured data points. Furthermore, attempts to impose structure (for example through forms and checklists) tend to be unpopular, run counter to normal record-keeping for communication and medico-legal purposes, and are difficult to sustain in the long term and outside specialist centres. An alternative approach is to derive data from text fields using natural language processing and thus enhance the structure of the record and information available. This has been demonstrated to be achievable in the mental health record through a range of research applications, particularly using the CRIS data resource at SLaM^21–29^ and creates at least a potential for improving predictive algorithms for use in routine clinical care.

Among the many potential applications of novel high-density data in clinical practice, the better prediction of service outcomes is an obvious candidate, and this was what we sought to evaluate in the studies reported here. The three outcomes were developed and evaluated over several years of stakeholder discussions and analyses and thus have some differences in their application, although all used a common approach to modelling and validation, and we feel that they are best considered together than as separate publications. The use of clinical data for service outcome prediction has a long history and are generally based on either small samples from individual services, or predictions are based on data limited in scope. The limitation with pre-EHR predictive modelling is that data are inevitably limited to measurements collected for a given cohort and therefore chosen in advance. Digital health records create at least the flexibility of information repositories within which models can be developed and optimised without the need for repeated data collection, as well as the potential for independent validation – across time periods, as adopted in our analyses, or between service providers.

### Interpretation of our findings

A key finding from the three outcomes evaluated was of differences in the degree of predictive model fit that could be achieved in development samples, as well as in the degree to which this translated into adequate prediction in validation samples. Although mental health in-patient outcomes were obvious targets for evaluating predictive modelling approaches, in retrospect it is not surprising that model performances were poor. Duration of in-patient episodes are strongly determined by external factors beyond the circumstances of the hospital admission episode or the clinical characteristics of the person experiencing it. These are likely to have included availability of housing and post-discharge support, which were not captured in the models and might indeed be unlikely to be recorded in a routine EHR without specific extra data collection. This and the salience of Mental Health Act status as a predictor might account for the relatively low fit and AUC for the development model, as well as the lack of prediction in the validation set. Although better development model fit was achieved for predicting readmission, compared with that for extended hospital admission, the algorithm derived from one year's data did not perform well in predicting occurrences for the second year. This may reflect again the salience of external (for example post-discharge) events that were not captured and did not generalise.

The better performance of a model built to predict high-cost service use may reflect the shorter time interval evaluated, as well as perhaps a more homogeneous set of records for deriving predictors, and an outcome less influenced by external, non-recorded factors. Indicators of early intensive service use were key variables in predicting higher costs, which is consistent with other studies.^30–32^ One limitation is the circularity of patients being more likely to receive greater service input if they have already received it previously (for example commencement of psychological therapy in the prediction window naturally predicting its continuation after the index date). There may also be confounding by the disorder severity influencing the level of staff contact. The negative prediction for patients referred from the criminal justice system is interesting because it suggests that these patients have less input from mental healthcare services perhaps because much of their contact occurs within the legal/criminal justice system. It is of interest that cannabis use was a negative predictor, which is inconsistent with other studies but this could be because this sample reflected all patients rather than just patients with psychosis.^22^

As described, an impetus behind our evaluations of predictive model performance was the novel possibility of including data from text fields that would not otherwise have been available and which we felt was likely to be clinically salient. This particularly applied to the clinical phenotype as quantified by symptoms recorded, as well as medication use. Importantly, it should be borne in mind that potential contributions to predictive models are likely to have been underestimated: both because of limitations in the natural language processing-derived constructs extracted (such as incomplete symptomatic characterisation), as well as the limited nuancing of information that was extracted (such as timing and intensity of symptoms, medication pathways, levels of adherence to treatments and insight into the underlying disorder) and information potentially amenable to natural language processing but not yet captured in CRIS (such as comorbidities, social support, lifestyle, stressful experiences). Despite these shortcomings, the inclusion of natural language processing-derived data increased the predictability in development models across all clinical outcomes explored, ranging between 12 and 46%, which might be because of the ability to capture more nuanced features of clinical presentation that cannot be captured using only structured data. This finding is encouraging and offers the potential to further exploit the large volumes of free-text data held within EHR when more sophisticated techniques are developed that could lead to improvements in predictability.

### Strengths

A strength of this study was the large sample and the diverse range of patient characteristics as the CRIS model provides ‘real-world’ and ‘real-time’ information on routine mental healthcare as it draws on large amounts of anonymised free-text information from the EHR, which enables analysis to be both large and deep as previously described.^33^ In addition, we were able to compare approaches across three different outcomes. We have also demonstrated that natural language processing techniques play a potentially important role in accessing previously unavailable data within the free text of the clinical record, which within mental health services is a substantial source of important clinical information. One key advantage to our approach, considering potential future applicability, is there was no additional ‘data entry’ required by clinical staff. Furthermore, the validity of the approaches was evaluated in routine mental healthcare rather than in an artificial research environment.

### Limitations

Considering potential limitations, this study was based at a single-site design within a centre of excellence, and at least some findings might reflect local issues and service provision; thus, replicability needs further investigation. However, in this respect, there was a conscious effort to ensure, where possible, the design decisions (for example choices of predictor variables) were potentially generalisable. The pragmatic decision to use first presentations within a single year as a development set and an independent year as a validation set is probably a conservative approach as the results could be improved if a split year approach was adopted. The development of the natural language processing-derived data presented here uses relatively simple natural language processing techniques and has provided increased depth to the EHR, however, this could be enhanced further as technology improves employing techniques such as machine learning. The data sources within our EHR are predominantly heterogeneous, which enables them to be applicable to a wide range of clinical services/specialities but a limitation exists regarding metadata in relation to the specific form/area of use. If this was more specific then the development of natural language processing applications tailored to the relevant areas of the EHR could provide additional benefits.

### Further directions for research

Considering future work, there is a need for further exploration of what performance levels might be achieved with additional and/or enhanced data such as linked primary care data or extending the development samples with an extended period for key variables such as intensity or duration of care prior to the 12-month window used here, as well as further scoping of requirements for this type of initiative, considering the potential demand/financial benefits it could offer – for example utilisation for more common conditions such as depression could result in greater cost savings. Anecdotally clinicians have suggested that false positives should be minimised at the expense of coverage of true positives but this aspect of implementation would need to be further explored in the context of an implementation programme. Furthermore, there may be costs associated with such algorithms in terms of processing capacity and decisions to be made on the frequency and timing of feedback and the way in which this is communicated. Following on from this, an important consideration is how to use predictive algorithms of this sort. In particular, if an outcome such as high service cost can be predicted with reasonable accuracy, it needs to be paired with a programme of actions to be taken and decisions to be made – for example, the high future service cost may be justified by the nature of the mental disorder in question, although it might indicate unmet need that could prompt earlier intervention.^34^ Similarly, it is important to ensure that those identified as at lower risk of a given adverse outcome are not disadvantaged and placed at higher risk of other outcomes not yet measured or evaluated.

Redesigned care pathways and other interventions clearly in turn require considerable further evaluation, taking into account pathway/treatment adherence and other outcome modifiers. On the other hand, a more immediate and less controversial application might be for services to evaluate at a group-level the distribution of referrals who are likely to require higher levels of intervention in the future, and to ensure that resources are allocated accordingly between teams.

## Supplementary material

For supplementary material accompanying this paper visit http://dx.doi.org/10.1192/bjo.2019.96. click here to view supplementary material
