# Supplementary material for: Predicting high-cost care in a mental health setting
Source: BJPsych Open. 2020 Jan 17;6(1):e10. doi: 10.1192/bjo.2019.96 (PMC7001466; doi:10.1192/bjo.2019.96)
Supplement: Supplementary file 1 [file S2056472419000966sup001.zip › S2056472419000966sup001/Supplementary Table 1.docx]

|  |
| --- |

| **Supplementary table 1:** Description of coefficients – predicting extended duration of hospital admission | | |
| --- | --- | --- |
|  |  |  |
| **Field type** | **Variable group** | **Variables** |
| NLP derived | Catatonia | Catalepsy**^1^**, echolalia**^4^**, echopraxia**^6^**, immobile**^4^**, mannerism**^1^**, mutism**^6^**, perseverance**^4^**, posturing**^1^**, rigidity**^4^**, stupor**^6^** and waxy flexibility**^2^** |
|  | Disorganised | Abstract thinking**^4^**, anhedonia**^1^**, circumstantial speech**^6^**, derailment**^4^**, flight of ideas**^6^**, formal thought disorder**^6^**, reduced coherence**^4^**, tangential speech**^1^** and thought block**^4^** |
|  | Manic | Disturbed sleep**^6^**, elation**^4^**, elevated mood**^6^**, euphoria**^6^**, grandiosity**^4^**, insomnia**^1^**, irritability**^4^** and pressured speech**^1^** |
|  | Prescribed medication | Antipsychotics^6^, clozapine^5^, **medication non-compliance** ^4^, f**irst generation antipsychotic (depot)**^4^, **first generation antipsychotic (not depot)**^4^, second **generation antipsychotic (depot)**^4^ and second **generation antipsychotic** not depot^6^ |
|  | Mood | Affective instability**^1^**, emotional instability**^1^** and mood instability**^4^** |
|  | Negative symptoms | Apathy**^1^**, blunted/flat affect**^4^**, emotional withdrawal**^4^**, poor rapport**^4^**, poverty of speech**^1^**, poverty of thought**^4^** and social withdrawal**^4^** |
|  | Positive symptoms | Aggression**^6^**, agitation**^6^**, arousal**^4^**, delusions**^6^**, hallucinations**^4^**, hostility**^4^**, paranoia**^4^** and persecutory ideas**^1^** |
| Structured | Current admission | Admission source**^6^**, admission service type**^4^** and MHA status**^6^** |
|  | Demographic/patient | Age**^1^**, days since last diagnosis**^4^**, deprivation group**^1^**, diagnosis**^6^**, ethnicity**^1^**, gender**^4^**, lives with**^4^** and marital status**^4^** |
|  | Health of nation outcome scale (HoNOS) | Days since HoNOS**^4^**, agitated behaviour**^3^**, cognitive problems**^3^**, daily living problems**^1^**, depressed mood**^4^**, hallucinations**^6^**, living conditions**^4^**, occupational problems**^4^**, other mental health problems**^4^**, physical illness**^4^**, problem drinking/drugs**^4^**, relationship problems**^6^** and self-injury**^4^** |
|  | Previous service use | Care co-ordinators(n)**^6^**, character count**^6^**, contact days**^4^**, care coordinator contact days**^5^**, care coordinator professional group**^4^**, days since discharge**^6^**, number of attended appointments**^1^**, previous admissions over 7 days**^4^**, previous admissions**^4^**, previous admissions average duration**^4^** and previous admissions over 7 days average duration**^4^** |
| **1** removed at univariate regression, **2** removed as standardised residuals > 3, **3** removed due to high correlation, **4** variables entered but not output by multivariate regression, **5** removed due to inflated standard error and **6** predictors of a higher duration of hospitalisation | | |
