# Supplementary material for: Predicting high-cost care in a mental health setting
Source: BJPsych Open. 2020 Jan 17;6(1):e10. doi: 10.1192/bjo.2019.96 (PMC7001466; doi:10.1192/bjo.2019.96)
Supplement: Supplementary file 1 [file S2056472419000966sup001.zip › S2056472419000966sup001/Supplementary Table 2.docx]

| **Supplementary table 2:** Description of coefficients – predicting hospital re-admission | | |
| --- | --- | --- |
|  |  |  |
| **Field type** | **Variable group** | **Variables** |
| NLP derived | Catatonia | Catalepsy^1^, echolalia^3^, echopraxia^1^, immobile^1^, mannerism^3^, mutism^3^, perseverance^3^, posturing^3^, rigidity^3^, stupor^1^ and waxy flexibility^1^ |
|  | Disorganised | Abstract thinking^3^, anhedonia^1^, circumstantial speech^1^, concentration^5^, derailment^3^, flight of ideas^1^, formal thought disorder^1^, guilt^1^, helpless^1^, hopeless^1^, low mood^1^, psychomotor^1^ reduced apetite^5^, reduced coherence^3^, lowered energy^1^, tangential speech^1^ , tearfulness^3^, thought block^1^, weight loss^3^ and worthlessness^5^ |
|  | Manic | Disturbed sleep^1^, elation^1^, elevated mood^5^, euphoria^1^, grandiosity^5^, insomnia^1^, irritability^1^ and pressured speech^1^ |
|  | Mood | Affective instability^3^, emotional instability^1^ and mood instability^5^ |
|  | Negative symptoms | Apathy^1^, blunted/flat affect^1^, emotional withdrawal^3^, motivation^1^, poor rapport^1^, poverty of speech^1^, poverty of thought^1^ and social withdrawal^5^ |
|  | Prescribed medication | Antipsychotics^3^, clozapine^5^, medication non-compliance^3^, first generation antipsychotic (depot)^5^, first generation antipsychotic (not depot)^3^, second generation antipsychotic (depot)^5^ and second generation antipsychotic not depot^5^ |
|  | Positive symptoms | Aggression^5^, agitation^1^, arousal^1^, delusions^5^, hallucinations^5^, hostility^3^, paranoia^3^ and persecutory ideas^1^ |
|  | Recreational drug use | Cannabis use^5^ |
| Structured | Index admission | Admission method**^1^**, admission source**^3^**, admission ward service type**^3^**, day of discharge**^1^**, duration of hospitalisation**^1^**, discharge destination^5^ , discharge method**^5^**, discharge ward service type**^3^**, MHA status (admission)^1^, MHA section 17^1^, MHA section 62^1^, borough of referring team**^5^**, rehabilitation ward flag^1^  and ward stay count^1^ |
|  | Demographic/patient | Age at index admission^3^, days since last diagnosis**^1^**, deprivation group^3^, diagnosis^5^, drug and alcohol^1^, ethnicity^1^, gender^1^, lives with^3^ and marital status^3^ |
|  | HoNOS | Days since HoNOS**^5^**, agitated behaviour**^3^**, cognitive problems**^3^**, daily living problems**^3^**, depressed mood**^3^**, hallucinations**^3^**, living conditions**^1^**, occupational problems**^5^**, other mental health problems**^3^**, physical illness**^1^**, problem drinking/drugs**^3^**, relationship problems**^3^** and self-injury**^3^** |
|  | Service use | Care co-ordinators(n)^1^, care coordinator duration^3^, care coordinator profession^1^, community contact days^3^, number emergency admissions^5^, inpatient admission count**^3^** and average duration admissions^5^ |
| **1** removed at univariate regression, **2** removed due to high correlation, **3** variables entered but not output by multivariate regression, **4** removed due to inflated standard error and **5** predictors of hospital re-admission | | |
