# Supplementary material for: Predicting high-cost care in a mental health setting
Source: BJPsych Open. 2020 Jan 17;6(1):e10. doi: 10.1192/bjo.2019.96 (PMC7001466; doi:10.1192/bjo.2019.96)
Supplement: Supplementary file 1 [file S2056472419000966sup001.zip › S2056472419000966sup001/Supplementary Table 3.docx]

| **Supplementary table 3:** Description of coefficients – predicting high service cost following first referral | | |
| --- | --- | --- |
|  | | |
| **Field type** | **Variable group** | **Variables** |
| NLP derived | Catatonia | Catalepsy^1^, echolalia^3^, echopraxia^3^, immobile^1^, mannerism^3^, mutism^3^, perseverance^1^, posturing^1^, rigidity^5^, stupor^3^ and waxy flexibility^3^ |
|  | Disorganised | Abstract thinking^3^, anhedonia^3^, circumstantial speech^3^, concentration^5^, derailment^3^, flight of ideas^5^, formal thought disorder^3^, low mood^5^, reduced apetite^5^, reduced coherence^1^, lowered energy^5^, tangential speech^1^ , tearfulness^5^, thought block^3^, weight loss^1^ and worthlessness^1^ |
|  | Manic | Disturbed sleep^5^, elation^3^, elevated mood^3^, euphoria^3^, grandiosity^3^, insomnia^3^, irritability^1^ and pressured speech^3^ |
|  | Mood | Affective instability^1^, emotional instability^1^ and mood instability^3^ |
|  | Negative symptoms | Apathy^3^, blunted/flat affect^3^, emotional withdrawal^1^, motivation^5^, poor rapport^3^, poverty of speech^3^, poverty of thought^3^ and social withdrawal^1^ |
|  | Positive symptoms | Aggression^5^, agitation^5^, arousal^1^, delusions^3^, hallucinations^5^, hostility^5^, paranoia^3^ and persecutory ideas^3^ |
| Structured | Demographic/patient | Age at referral acceptance ^5^, deprivation group^3^, diagnosis^5^, ethnicity^3^, gender^4^, harm/neglect^3^, lives with^3^, marital status^5^, next of kin^5^ and safeguarding^3^ |
|  | Service use | Change of address^3^, attended appointments(consultant)^5^, attended appointments(staff grade medic)^3^, attended appointments (nursing)^5^, attended appointments(OT)^3^, attended appointments(Other therapist)^3^, attended appointments (psychology)^5^, attended appointments(social worker)^3^, care co-ordinators(n)^1^, clinical academic group (CAG)^5^ , community contact days^5^, care coordinator profession^5^, emergency appointments^1^, emergency inpatient admission count^3^, consultant recorded^3^, consultant speciality^5^, inpatient admission count^5^, inpatient bed days^5^, GP practice(n)^3^, GP recorded^1^, HoNOS recorded^3^, IAPT contact^1^, IAPT sessions^1^, number of teams^5^, referral priority^3^, referral source^5^, referral status^3^, risk screen recorded^3^ and temporary address count^3^ |
| **1** removed at univariate regression, **2** removed due to high correlation, **3** variables entered but not output by multivariate regression, **4** removed due to inflated standard error and **5** predictors of high total service cost | | |
