# Supplementary material for: Predicting high-cost care in a mental health setting
Source: BJPsych Open. 2020 Jan 17;6(1):e10. doi: 10.1192/bjo.2019.96 (PMC7001466; doi:10.1192/bjo.2019.96)
Supplement: Supplementary file 1 [file S2056472419000966sup001.zip › S2056472419000966sup001/Supplementary Table 4.docx]

**Supplementary Table 4:** Characteristics of samples used to predict extended duration of hospitalisation

|  | **Development** | **Validation** | **X^2^ (T value)** | **P** |
| --- | --- | --- | --- | --- |
| Number of first presentations | 808 | 760 |  |  |
| **Age**, mean (standard deviation) | 39.3, (11.2) | 39.4, (12.0) | (0.170) | 0.870 |
| **Gender** |  |  |  |  |
| Female | 332, (41.1) | 306, (40.3) | 0.104 | 0.747 |
| Male | 476, (58.9) | 454, (59.7) | 0.104 | 0.747 |
| **Marital status** |  |  |  |  |
| Divorced/Single | 731, (90.5) | 692, (91.1) | 0.169 | 0.681 |
| Married/Cohabiting | 77, (9.5) | 68, (8.9) | 0.169 | 0.681 |
| **Ethnic group** |  |  |  |  |
| Asian | 41, (5.1) | 42, (5.5) | 0.125 | 0.724 |
| Black | 476, (58.9) | 425, (55.9) | 1.441 | 0.230 |
| Mixed | 26, (3.2) | 34, (4.5) | 1.796 | 0.180 |
| Other | 37, (4.6) | 36, (4.7) | 0.009 | 0.925 |
| White | 228, (28.2) | 223, (29.3) | 0.231 | 0.631 |
| **Diagnostic group** |  |  |  |  |
| Bipolar disorder | 155, (19.2) | 162, (21.3) | 1.070 | 0.301 |
| Non Affective psychosis | 653, (80.8) | 598, (78.7) | 1.070 | 0.301 |
| **Legal status of admission** |  |  |  |  |
| Informal | 340, (42.1) | 310, (40.8) | 0.273 | 0.602 |
| MHA Section 2 | 294, (36.4) | 307, (40.4) | 2.649 | 0.104 |
| MHA Section 3 | 174, (21.5) | 143, (18.8) | 1.771 | 0.183 |

Numbers and (percentages) presented unless otherwise stated

Significance levels for categorical variables are determined using the N-1 Chi-squared test and paired T- test for means
