# Supplementary material for: Predicting high-cost care in a mental health setting
Source: BJPsych Open. 2020 Jan 17;6(1):e10. doi: 10.1192/bjo.2019.96 (PMC7001466; doi:10.1192/bjo.2019.96)
Supplement: Supplementary file 1 [file S2056472419000966sup001.zip › S2056472419000966sup001/Supplementary Table 5.docx]

**Supplementary table 5:** individual contribution to the extended hospitalisation duration prediction

| **Variable** | **Z score** | **OR** | **95% CI** |
| --- | --- | --- | --- |
| Mental health act status: section 3 | 5.68 | 4.05 | 2.51 - 6.60 |
| Symptom: formal thought disorder | 3.19 | 1.03 | 1.01 - 1.05 |
| Admission source: non NHS hospital | 2.83 | 3.11 | 1.40 - 6.80 |
| Character count | 2.49 | 1.00 | 1.00 - 1.00 |
| Number of antipsychotic medications | 2.49 | 1.19 | 1.04 - 1.37 |
| Symptoms: disturbed sleep | 2.31 | 1.03 | 1.00 - 1.06 |
| Symptom: catatonia stupor | 1.98 | 2.58 | 1.08 - 7.84 |
| Admission source: criminal Justice | 1.96 | 2.16 | 0.99 - 4.65 |
| HoNOS hallucination | 1.73 | 1.00 | 1.00 - 1.00 |
| Care coordinator count | 1.71 | 1.20 | 0.97 - 1.47 |
| Symptom: aggression | 1.68 | 1.01 | 1.00 - 1.02 |
| Symptom: mutism | 1.51 | 1.01 | 1.00 - 1.03 |
| Symptom: elevated mood | 1.44 | 1.17 | 0.94 - 1.44 |
| Admission source: other NHS trust | 1.15 | 1.45 | 0.76 - 2.72 |
| Days Since last discharge: not discharged previous 12 months | 0.88 | 1.35 | 0.70 - 2.66 |
| Mental health act status: section 2 | 0.86 | 1.23 | 0.77 - 1.96 |
| Admission source: usual residence | 0.79 | 1.21 | 0.76 - 1.92 |
| Days since last discharge: between 34 and 102 | 0.59 | 1.21 | 0.64 - 2.31 |
| Days Since last discharge: between 214 and 36 | 0.05 | 1.02 | 0.51 - 2.02 |
| Admission source: other | -0.28 | 0.85 | 0.23 - 2.51 |
| Symptom: agitation | -1.53 | 0.99 | 0.97 - 1.00 |
| Symptoms: euphoria | -1.62 | 0.22 | 0.03 - 0.97 |
| Days Since last discharge: between 103 and 213 | -1.94 | 0.49 | 0.24 - 1.00 |
| HoNOS relationship problems | -1.96 | 1.00 | 1.00 - 1.00 |
| Diagnosis: non affect psychosis | -2.08 | 0.61 | 0.39 - 0.98 |
| Symptoms: delusions | -2.09 | 0.99 | 0.98 - 1.00 |
| Number of second generation antipsychotic (not depot) medications | -2.67 | 0.72 | 0.56 - 0.92 |
| Symptom: circumstantial | -3.15 | 0.90 | 0.84 - 0.96 |
| Symptom: flight of ideas | -3.54 | 0.76 | 0.64 - 0.87 |
