# Supplementary material for: Predicting high-cost care in a mental health setting
Source: BJPsych Open. 2020 Jan 17;6(1):e10. doi: 10.1192/bjo.2019.96 (PMC7001466; doi:10.1192/bjo.2019.96)
Supplement: Supplementary file 1 [file S2056472419000966sup001.zip › S2056472419000966sup001/Supplementary Table 6.docx]

**Supplementary Table 6:** Characteristics of samples used to predict hospital readmission

|  | **Development** | **Validation** | **X^2^ (T value)** | **P** |
| --- | --- | --- | --- | --- |
| Number of discharges | 1,650 | 1,569 |  |  |
| **Age**, mean (standard deviation) | 39.8, (11.7) | 39.6, (12.0) | (-0.479) | 0.632 |
| **Gender** |  |  |  |  |
| Female | 738, (44.7) | 666, (42.4) | 1.730 | 0.188 |
| Male | 912, (55.3) | 903, (57.6) | 1.730 | 0.188 |
| **Marital status** |  |  |  |  |
| Divorced/Single | 1,448, (87.8) | 1386, (88.3) | 0.191 | 0.662 |
| Married/Cohabiting | 202, (12.2) | 183, (11.7) | 0.191 | 0.662 |
| **Ethnic group** |  |  |  |  |
| Asian | 88, (5.3) | 78, (5.0) | 0.148 | 0.700 |
| Black | 855, (51.8) | 795, (50.7) | 0.389 | 0.533 |
| Mixed | 58, (3.5) | 57, (3.6) | 0.023 | 0.878 |
| Other | 64, (3.9) | 84, (5.4) | 4.096 | **0.043** |
| White | 584, (35.4) | 555, (35.4) | 0.000 | 1.000 |
| **Diagnostic group** |  |  |  |  |
| Bipolar disorder | 407, (24.7) | 444, (28.3) | 5.355 | **0.021** |
| Non Affective psychosis | 969, (58.7) | 889, (56.7) | 1.318 | 0.251 |
| Other | 274, (16.6) | 239, (15.0) | 1.546 | 0.214 |
| **Legal status of admission** |  |  |  |  |
| Informal | 726, (44.0) | 669, (42.6) | 1.441 | 0.230 |
| Formal admission | 924, (56.0) | 900, (57.4) | 0.642 | 0.423 |

Numbers and (percentages) presented unless otherwise stated

Significance levels for categorical variables are determined using the N-1 Chi-squared test and paired T- test for means
