# Supplementary material for: Predicting high-cost care in a mental health setting
Source: BJPsych Open. 2020 Jan 17;6(1):e10. doi: 10.1192/bjo.2019.96 (PMC7001466; doi:10.1192/bjo.2019.96)
Supplement: Supplementary file 1 [file S2056472419000966sup001.zip › S2056472419000966sup001/Supplementary Table 7.docx]

**Supplementary table 7:** individual contribution to the hospitalisation readmission prediction

| **Variable** | **Z score** | **OR** | **95% CI** |
| --- | --- | --- | --- |
| **Number emergency admissions** | 3.52 | 1.93 | 1.33 - 2.78 |
| **Clozapine during inpatient stay** | 2.72 | 2.30 | 1.24 - 4.12 |
| **Second generation antipsychotic (not depot)** | 2.10 | 1.28 | 1.02 - 1.62 |
| Average duration admissions | 2.08 | 1.01 | 1.00 - 1.02 |
| **Medication non-compliance** | 1.92 | 1.05 | 1.00 - 1.10 |
| **First generation antipsychotic ( depot)** | 1.85 | 1.49 | 0.96 - 2.24 |
| **Second generation antipsychotic (depot)** | 1.78 | 1.52 | 0.95 - 2.40 |
| **Symptom: aggression** | 1.61 | 7.02 | 0.58 - 67.5 |
| **Discharge destination: private sector** | 1.09 | 1.73 | 0.63 - 4.58 |
| **Discharge destination: external** | 0.94 | 1.68 | 0.55 - 4.90 |
| **Discharge method: other** | 0.78 | 2.48 | 0.27 - 30.01 |
| **Diagnosis: other psychosis** | -0.13 | 0.95 | 0.46 - 1.93 |
| **Discharge method: on professional advice** | -0.66 | 0.52 | 0.09 - 4.81 |
| **Days since HoNOS recorded** | -1.24 | 1.00 | 0.99 - 1.00 |
| **HoNOS occupational problems** | -1.25 | 1.00 | 1.00 - 1.00 |
| **Discharge destination: home treatment team** | -1.34 | 0.37 | 0.07 - 1.40 |
| **Symptom: worthless** | -1.40 | 0.00 | 0.00 - 0.01 |
| **Symptom: appetite** | -1.41 | 0.00 | 0.00 - 2.02 |
| **Symptom: hallucinations** | -1.41 | 0.19 | 0.01 - 1.57 |
| **Symptom: concentration** | -1.41 | 0.00 | 0.00 - 5.06 |
| **Symptom: social withdrawal** | -1.52 | 0.00 | 0.00 - 0.73 |
| **Symptom: coherence** | -1.56 | 0.01 | 0.00 - 1.87 |
| **Symptom: elevated mood** | -1.57 | 0.00 | 0.00 - 0.13 |
| **Cannabis use** | -1.93 | 0.98 | 0.95 - 1.00 |
| Borough of referring team**: Lambeth** | -1.94 | 0.36 | 0.13 - 1.07 |
| **Symptom: delusions** | -1.96 | 0.05 | 0.00 - 0.79 |
| **Symptom: grandiosity** | -1.98 | 0.00 | 0.00 - 0.39 |
| **Symptom: mood instability** | -2.15 | 0.00 | 0.00 - 0.11 |
| Borough of referring team**: Croydon** | -2.23 | 0.28 | 0.09 - 0.90 |
| **Discharge method: internal** | -2.27 | 0.06 | 0.00 - 0.75 |
| Borough of referring team**: Lewisham** | -2.35 | 0.27 | 0.09 - 0.85 |
| **Diagnosis: non affective psychosis** | -2.80 | 0.45 | 0.26 - 0.79 |
| **Discharge destination: GP** | -2.97 | 0.35 | 0.18 - 0.70 |
| Borough of referring team**: Southwark** | -3.11 | 0.18 | 0.06 - 0.56 |
| **Discharge destination: other** | -4.90 | 0.19 | 0.10 - 0.37 |
