# Supplementary material for: Predicting high-cost care in a mental health setting
Source: BJPsych Open. 2020 Jan 17;6(1):e10. doi: 10.1192/bjo.2019.96 (PMC7001466; doi:10.1192/bjo.2019.96)
Supplement: Supplementary file 1 [file S2056472419000966sup001.zip › S2056472419000966sup001/Supplementary Table 8.docx]

**Supplementary Table 8:** Characteristics of samples used to predict total service cost

|  | **Development** | **Validation** | **X^2^(T value)** | **P** |
| --- | --- | --- | --- | --- |
| Number of first presentations | 4,494 | 4,675 |  |  |
| **Age**, mean (standard deviation) | 49.3, (22.5) | 48.1, (22.6) | (-2.547) | **0.011** |
| **Gender** |  |  |  |  |
| Female | 2,552, (56.8) | 2,648, (56.6) | 0.037 | 0.847 |
| Male | 1,492, (43.2) | 2,027, (43.4) | 0.037 | 0.847 |
| **Marital status** |  |  |  |  |
| Divorced/Single | 2,937, (65.4) | 3,000, (64.2) | 1.446 | 0.229 |
| Married/Cohabiting | 1,277, (28.4) | 1,246, (26.7) | 3.319 | 0.069 |
| Unknown | 280, (6.2) | 429, (9.2) | 28.911 | **0.000** |
| **Ethnic group** |  |  |  |  |
| Asian | 262, (5.8) | 243, (5.2) | 1.589 | 0.208 |
| Black | 722, (16.1) | 709, (15.2) | 1.406 | 0.236 |
| Mixed | 94, (2.1) | 82, (1.8) | 1.080 | 0.299 |
| Other | 440, (9.8) | 504, (10.8) | 2.478 | 0.116 |
| White | 2,750, (61.2) | 2,748, (58.8) | 5.498 | **0.019** |
| Unknown | 226, (5.0) | 389, (8.3) | 40.010 | **0.000** |
| **Diagnostic group** |  |  |  |  |
| Bipolar disorder | 147, (3.3) | 165, (3.5) | 0.279 | 0.597 |
| Eating disorder | 182, (4.0) | 183, (3.9) | 0.060 | 0.806 |
| Mood/Anxiety disorder | 1,080, (24.0) | 1,169, (25.0) | 1.238 | 0.266 |
| Organic | 932, (20.7) | 781, (16.7) | 24.151 | **0.000** |
| Personality disorder | 96, (2.1) | 131, (2.8) | 4.684 | **0.030** |
| Schizophrenia | 337, (7.5) | 346, (7.4) | 0.033 | 0.855 |
| Substance misuse | 84, (1.9) | 55, (1.2) | 7.389 | **0.007** |
| Other diagnosis | 487, (10.8) | 538, (11.5) | 1.133 | 0.287 |
| Not recorded | 1,149, (25.6) | 1,307, (28.0) | 6.723 | **0.010** |

Numbers and (percentages) presented unless otherwise stated

Significance levels for categorical variables are determined using the N-1 Chi-squared test and paired T- test for means
