# Supplementary material for: Predicting high-cost care in a mental health setting
Source: BJPsych Open. 2020 Jan 17;6(1):e10. doi: 10.1192/bjo.2019.96 (PMC7001466; doi:10.1192/bjo.2019.96)
Supplement: Supplementary file 1 [file S2056472419000966sup001.zip › S2056472419000966sup001/Supplementary Table 9.docx]

**Supplementary table 9:** individual contribution to the total service cost prediction

| **Variable** | **z score** | **OR** | **95% CI** |
| --- | --- | --- | --- |
| Inpatient bed days | 8.45 | 1.04 | 1.03 - 1.05 |
| Community contact days | 6.35 | 1.11 | 1.08 - 1.15 |
| Inpatient admissions | 3.62 | 2.19 | 1.43 - 3.35 |
| Clinical psychology contacts | 3.52 | 1.10 | 1.04 - 1.16 |
| Service type: psychosis | 3.42 | 4.78 | 2.01 - 12.16 |
| Symptom: low mood | 3.18 | 7.17 | 2.03 - 23.45 |
| Service type: mood, anxiety and personality | 3.15 | 3.77 | 1.71 - 9.01 |
| First clinical document: ICD10 | 2.87 | 1.90 | 1.22 - 2.94 |
| Care coordinator profession: nurse | 2.85 | 3.89 | 1.65 - 10.99 |
| Care coordinator profession: other | 2.64 | 3.93 | 1.51 - 11.83 |
| Care coordinator profession: no care coordinator | 2.55 | 3.28 | 1.42 - 9.09 |
| Service type: MHOA and dementia | 2.06 | 2.79 | 1.07 - 7.63 |
| Next of kin/family recorded | 1.94 | 1.34 | 0.99 - 1.8 |
| Emergency contact | 1.83 | 1.50 | 0.97 - 2.31 |
| Care coordinator profession: consultant psychiatrist | 1.83 | 2.64 | 0.98 - 8.07 |
| Consultant contacts | 1.78 | 1.08 | 0.99 - 1.18 |
| Symptom: hallucinations | 1.69 | 2.27 | 0.83 - 5.57 |
| Service type: other | 1.55 | 2.34 | 0.81 - 6.99 |
| Care coordinator profession: occupational therapy | 1.54 | 2.41 | 0.82 - 7.86 |
| Care coordinator profession: social worker | 1.53 | 2.37 | 0.81 - 7.64 |
| First clinical document: event | 1.23 | 1.20 | 0.90 - 1.59 |
| Care coordinator profession: junior medical | 1.14 | 1.84 | 0.67 - 5.69 |
| Referral source: other clinical speciality | 1.08 | 1.37 | 0.77 - 2.44 |
| Diagnosis: eating disorder | 0.94 | 1.48 | 0.65 - 3.41 |
| First clinical document: other | 0.61 | 1.23 | 0.61 - 2.29 |
| Diagnosis: personality disorder | 0.61 | 1.30 | 0.56 - 2.98 |
| Service type: Psychological Medicine | 0.61 | 1.31 | 0.57 - 3.21 |
| Care coordinator profession: other therapist | 0.55 | 2.12 | 0.12 - 25.45 |
| Diagnosis: mood/anxiety | 0.40 | 1.13 | 0.63 - 2.11 |
| Diagnosis: substance misuse | 0.26 | 1.13 | 0.45 - 2.73 |
| Marital Status: single/divorced | 0.08 | 1.01 | 0.75 - 1.36 |
| Diagnosis: other | 0.03 | 1.01 | 0.48 - 2.18 |
| Referral source: other | 0.01 | 1.00 | 0.57 - 1.76 |
| Diagnosis: schizophrenia | -0.19 | 0.94 | 0.50 - 1.81 |
| Referral source: GP | -0.68 | 0.82 | 0.46 - 1.46 |
| Diagnosis: organic | -1.43 | 0.59 | 0.29 - 1.24 |
| Age at referral acceptance | -1.52 | 0.99 | 0.98 – 1.00 |
| Consultant recorded | -1.56 | 0.78 | 0.57 - 1.07 |
| Referral source: criminal justice | -1.57 | 0.49 | 0.20 - 1.19 |
| Accepted team episodes | -1.66 | 0.88 | 0.75 - 1.02 |
| Diagnosis: not recorded | -1.67 | 0.57 | 0.30 - 1.12 |
| Symptom: anergia | -1.72 | 0.00 | 0.00 - 0.05 |
| Symptom: disturbed sleep | -1.80 | 0.44 | 0.17 - 1.03 |
| Symptom: appetite | -1.87 | 0.13 | 0.01 - 0.85 |
| Symptom: tearful | -1.90 | 0.34 | 0.11 - 0.97 |
| Marital Status: unknown | -2.22 | 0.35 | 0.13 - 0.81 |
| Cannabis use | -2.26 | 0.96 | 0.93 - 0.99 |
| Nurse contacts | -2.27 | 0.95 | 0.92 - 0.99 |
| Symptom: concentration | -2.38 | 0.16 | 0.03 - 0.64 |
| Emergency contact count | -3.87 | 0.77 | 0.67 - 0.88 |
| Referral Status: discharged | -5.02 | 0.41 | 0.29 - 0.58 |
